# Neuroprotective Effects of Hesperidin, a Plant Flavanone, on Rotenone-Induced Oxidative Stress and Apoptosis in a Cellular Model for Parkinson's Disease

**DOI:** 10.1155/2013/102741

**Published:** 2013-09-24

**Authors:** Kuppusamy Tamilselvam, Nady Braidy, Thamilarasan Manivasagam, Musthafa Mohamed Essa, Nagarajan Rajendra Prasad, Subburayan Karthikeyan, Arokyasamy Justin Thenmozhi, Subash Selvaraju, Gilles J. Guillemin

**Affiliations:** ^1^Department of Biochemistry and Biotechnology, Faculty of Science, College Rd, Annamalai Nagar, Chidambaram, Tamil Nadu 608002, India; ^2^Centre for Healthy Brain Ageing, School of Psychiatry, Faculty of Medicine, University of New SouthWales, Sydney 2031, Australia; ^3^Department of Food Science and Nutrition, College of Agriculture and Marine Sciences, Sultan Qaboos University, P.O. Box 50, Muscat 123, Oman; ^4^Neuropharmacology Group, MND and Neurodegenerative Diseases Research Centre, Australian School of Advanced Medicine, Macquarie University, Balaclava Road, North Ryde, Sydney, NSW 2109, Australia

## Abstract

Rotenone a widely used pesticide that inhibits mitochondrial complex I has been used to investigate the pathobiology of PD both *in vitro* and *in vivo*. Studies have shown that the neurotoxicity of rotenone may be related to its ability to generate reactive oxygen species (ROS), leading to neuronal apoptosis. The current study was carried out to investigate the neuroprotective effects of hesperidin, a citrus fruit flavanol, against rotenone-induced apoptosis in human neuroblastoma SK-N-SH cells. We assessed cell death, mitochondrial membrane potential, ROS generation, ATP levels, thiobarbituric acid reactive substances, reduced glutathione (GSH) levels, and the activity of catalase, superoxide dismutase (SOD) and glutathione peroxidase (GPx) using well established assays. Apoptosis was determined in normal, rotenone, and hesperidin treated cells, by measuring the protein expression of cytochrome c (cyt c), caspases 3 and 9, Bax, and Bcl-2 using the standard western blotting technique. The apoptosis in rotenone-induced SK-N-SH cells was accompanied by the loss of mitochondrial membrane potential, increased ROS generation, the depletion of GSH, enhanced activities of enzymatic antioxidants, upregulation of Bax, cyt c, and caspases 3 and 9, and downregulation of Bcl-2, which were attenuated in the presence of hesperidin. Our data suggests that hesperidin exerts its neuroprotective effect against rotenone due to its antioxidant, maintenance of mitochondrial function, and antiapoptotic properties in a neuroblastoma cell line.

## 1. Introduction

Parkinson's disease (PD) is the second most common neurodegenerative disorder after Alzheimer's disease. It is characterised by the progressive loss of dopaminergic neurons in the substantia nigra pars compacta and subsequent depletion of dopamine in the striatum, the main projection area of the substantia nigra. Numerous studies using postmortem human tissues, animal models, and neuronal cell lines have reported the involvement of several pathological mechanisms responsible for the loss of dopaminergic neurons in PD, including elevated levels of iron, ubiquitin-proteasome system (UPS) dysfunction and impairment, altered calcium homeostasis, excitotoxicity, inflammation, oxidative stress, and release of apoptotic factors [1, 2].

Rotenone is a naturally occurring lipophilic compound exhibiting insecticide-like properties and is obtained from the roots of certain plants species (*Derris* and *Lonchocarpus*) [3]. It is one of the common neurotoxic agents used to examine the development of PD in animal models [4] and induces similar toxicity in primary dopaminergic cultures derived from embryonic mouse mesencephalon [5], PC12 cells [6], and human neuroblastoma SH-SY5Y cells [7]. It inhibits mitochondrial electron transfer chain (ETC) complex I, which enhances the formation of ROS and leads to modest depletion of ATP and mitochondrial dysfunction culminating in apoptotic cell death [8].

Current pharmacological therapies for PD are inadequate, and alternative strategies such as stem cell therapy, neurotransplantation, and deep brain stimulation are still in infant stage. There has been considerable interest in the development of neuroprotective drugs from natural origins as a therapeutic strategy for PD [9]. Citrus fruits and their products are important sources of health-promoting constituents and are widely consumed around the world [10]. Hesperidin is a naturally occurring flavanone that exists in citrus and other plants and can be isolated in large amounts from the peels of *Citrus aurantium* (bitter orange), *Citrus sinensis* (sweet orange), and *Citrus unshiu* (satsuma mandarin) [11]. Hesperidin is reported to exert a wide range of pharmacological effects such as antioxidant, anti-inflammatory, antihypercholesterolemic, and anticarcinogenic properties [12]. It has also been demonstrated that hesperidin can protect neurons against various types of insults associated with many neurodegenerative diseases [13]. In this study, we investigated the neuroprotective effect of hesperidin on rotenone-induced cellular model for PD by analysing its effect on rotenone-mediated oxidative stress generation, mitochondrial dysfunction, and apoptosis in human neuroblastoma SK-N-SH cells.

## 2. Materials and Methods

### 2.1. Chemicals

Rotenone, hesperidin, thiobarbituric acid (TBA), phenazine methosulfate (PMS), nitroblue tetrazolium (NBT), 5,5-dithiobis(2-nitrobenzoic acid) (DTNB), 3-(4,5-dimethylthiazol-2-yl)-2,5-diphenyltetrazolium bromide (MTT), 2,7-diacetyl dichlorofluorescein (DCFH-DA), rhodamine 123 (Rh-123), heat-inactivated fetal calf serum (FCS), Dulbecco's modified Eagle's medium (DMEM), glutamine, penicillin-streptomycin, EDTA, and trypsin were purchased from Sigma Chemicals Co., St. Louis, USA. Anti-Bcl-2, anti-Bax, caspase 3, caspase 9, and cytochrome c antibodies were obtained from Cell Signalling (USA) and *β*-actin antibodies were purchased from Santa Cruz Biotechnology, Inc., USA. ATP Bioluminescence Assay Kit HS II was purchased from Roche Molecular Biochemicals. Anti-mouse and anti-rabbit secondary antibodies were purchased from Genei, Bangalore, India.

### 2.2. Cell Culture

The SK-N-SH neuroblastoma cell line was obtained from the National Centre for Cell Science (NCCS) Pune, India. Cells were grown in (DMEM) 12 (1 : 1), supplemented with 2 mM glutamine, penicillin (100 U/mL), streptomycin (100 U/mL), gentamicin (100 *μ*g/mL), and 10% (vol/vol) heat-inactivated fetal bovine serum (FBS) (Sigma Chemicals Co, St Louis, USA), and the medium was changed every two days. Cells were maintained at 37°C in CO_2_ incubator in a saturated humidity atmosphere containing 95% air and 5% CO_2_. Rotenone and hesperidin were dissolved in fresh DMSO (0.05%) prior to each experiment. Hesperidin was added 4 h prior to rotenone treatment.

In Experiment I, cells were incubated with different concentrations of rotenone (2.5, 5, 50, 100, and 200 nM) for 24 h, and MTT assay was performed to detect IC_50_ value of rotenone. In Experiment II, cells were pretreated with different concentrations of hesperidin (2.5, 5, 10, 20, and 40 *μ*g) for 4 h and then incubated with rotenone (effective dose) for 24 h. The effective dose of hesperidin was used to identify potential neuroprotective effects against rotenone toxicity.

### 2.3. MTT Assay

The proliferation of cells treated with various concentrations of rotenone and hesperidin was determined by the MTT assay based on the detection of mitochondrial dehydrogenase activity in living cells [14, 15]. MTT was added to each well, and the plates were incubated at 37°C for 4 h. Afterwards, the cells were centrifuged for 10 min, and the supernatant was removed, 200 *μ*L of DMSO was added into each well, and absorbance was measured in a microplate reader (Molecular Devices, CA, USA) at 595 nm.

### 2.4. Determination of Intracellular ROS Generation

The formation of ROS was measured by using a nonfluorescent probe, 2,7-diacetyl dichlorofluorescein (DCFH-DA) that can penetrate into the intracellular matrix of cells, where it is oxidized by ROS to form fluorescent dichlorofluorescein (DCF) [16]. The percentage of ROS was estimated in the control, hesperidin, and rotenone-treated SK-N-SH neuroblastoma cells. Briefly, an aliquot of the isolated cells 8 × 10^6^ cells/mL was made up to a final volume of 2 mL in normal PBS (pH 7.4). Then, 1 mL aliquot of cells was taken to which 100 *μ*L DCFH-DA (10 *μ*M) was added and incubated at 37°C for 30 min. Fluorescent measurements were made with excitation and emission filters were set at 485 ± 10 nm, and 530 ± 12.5 nm respectively (Shimadzu RF-5301 PC spectrofluorometer). All initial fluorescent values (time 0) were found to differ from each other by less than 5%. Results were expressed as percentage; increase in fluorescence was calculated using the formula [(*F*_*t*_30__ − *F*_*t*_0__)/(*F*_*t*_0__ × 100)], and the fluorescence intensities at 0 and 30 min were measured.

### 2.5. Measurement of Intracellular ATP Levels

Cells were collected by centrifugation, and intracellular ATP was measured with a luminometer using an ATP Bioluminescence Assay Kit HS II (Roche Molecular Biochemicals) according to the manufacturer's instructions.

### 2.6. TBARS Assay

SK-N-SH neuroblastoma cells were suspended in 130 mM KCl and 50 mM PBS containing 0.1 mL of 0.1 M dithiothreitol (DTT) and centrifuged at 20,000 g for 15 min (4°C). The supernatant was taken for biochemical estimation. The level of lipid peroxidation was determined by analysing TBARS as previously described [17]. The pink-coloured chromogen formed by the reaction of 2-TBA with breakdown products of lipid peroxidation was measured.

### 2.7. SOD Activity Assay

 Superoxide dismutase (SOD) activity was assayed by the method based on the inhibition of the formation of (NADH-PMS-NBT) complex as previously described [18].

### 2.8. Catalase Activity Assay

Catalase (CAT) activity was assayed by the decomposition of hydrogen peroxide as previously described [19]. A decrease in absorbance due to H_2_O_2_ degradation was monitored at 240 nm for 1 min.

### 2.9. Glutathione Peroxidase Activity Assay

The activity of glutathione peroxidase (GPx) was assayed spectrophotometrically as previously described [20]. Briefly, a known amount of enzyme preparation was allowed to react with hydrogen peroxides (H_2_O_2_) and GSH for a specified time period. The GSH content remaining after the reaction was measured.

### 2.10. Estimation of Glutathione

The total GSH content was measured by the method as previously described [21]. This method was based on the development of a yellow colour, when 5,5-dithiobis (2-nitrobenzoic acid) was added to compound containing sulfhydryl groups.

### 2.11. Changes in Mitochondrial Transmembrane Potential (Δ*ψm*)

The change in  Δ*ψm*  in different treatment groups was observed microscopically and determined fluorometrically using the fluorescent dye rhodamine 123 (Rh-123) as previously described [22]. Briefly, after incubation with treatment compounds for 24 h, 1 *μ*L of fluorescent dye Rh-123 (5 m mol/l) was added to the cells and returned to the incubator for 15 min [22]. The cells were then washed with PBS, observed under fluorescence microscope, and estimated by using blue filter (450–490 nm) (Olympus BX60 fluorescence microscope). Polarized mitochondria emit orange-red fluorescence, and depolarized mitochondria emit green fluorescence. The fluorescence intensity was measured at 535 nm using FLUOstar OPTIMA fluorometer (Durham, NC, USA).

### 2.12. Western Blotting

Briefly, cells seeded in 6-well plates were harvested and washed with PBS. Cells were lysed in 100 *μ*L lysis buffer containing 20 mM Tris-Hcl, pH 7.4, 150 mM NaCl, 1 mM EDTA, 30 *μ*g/mL aprotinin, and 1 mM phenylmethylsulfonyl fluoride and subjected to 12.5% polyacrylamide gel electrophoresis. A total volume of 30 *μ*g of protein was loaded per lane. The separated proteins were blotted onto a PVDF membrane by semidry transfer (Bio-Rad). After blocking with 5% nonfat milk in TBS, the membranes were then incubated with various antibodies: Bcl-2, Bax, caspases 3 and 9, cytochrome c, and *β*-actin. The following dilutions were used for Bcl-2 and Bax (1 : 500), cytochrome c, caspases 3 and 9 (1 : 1000), and *β*-actin (1 : 2000). After primary antibody incubation, the membranes were incubated with secondary antibody at a concentration of 1 : 2000. Then, the membranes were washed with Tris-buffered saline and 0.05% Tween 20 thrice for 10 min interval; after extensive washes in TBST, the bands were visualized by treating the membranes with 3,3′-diaminobenzidine tetrahydrochloride (western blot detection reagent, Sigma, USA). Densitometry was performed using “Image J” analysis software.

### 2.13. Data Analysis

Statistical analysis was performed using one-way analysis of variance followed by Duncan's multiple range test (DMRT) using Statistical Package for the Social Science (SPSS) software package version 12.0. Results were expressed as mean ± SD for four experiments in each group. *P*  values <0.05 were considered significant.

## 3. Results

### 3.1. Effect of Hesperidin on Rotenone-Induced Cell Death

**Figure 1:**
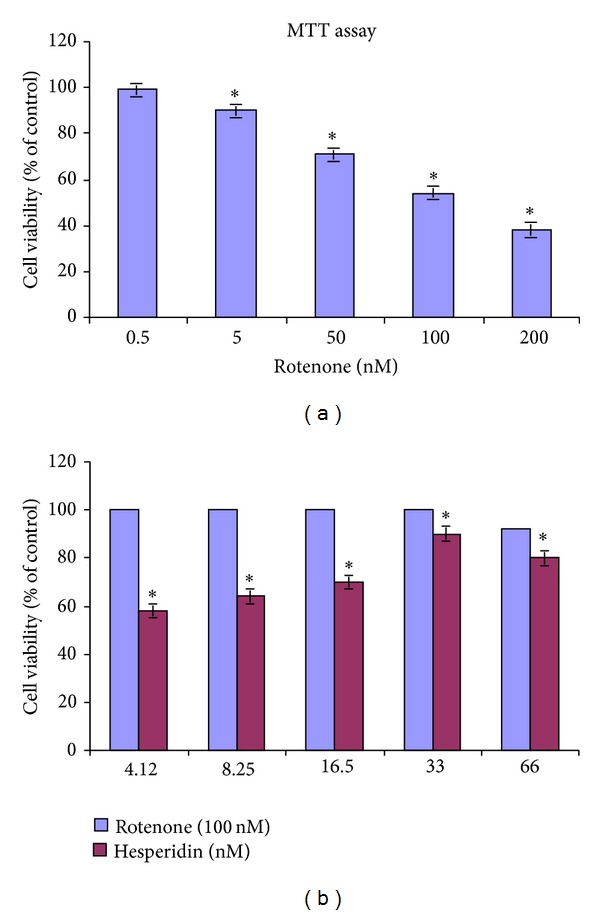
Effect of hesperidin on rotenone-induced reduction in cell proliferation in SK-N-SH neuroblastoma cells. (a) The dose-dependent effect of rotenone (0.5, 5, 50, 100, and 200 nM) on cell proliferation after 24 h. Values are presented as mean ± SD of four experiments in each group. 50% inhibition concentration value (IC_50_) was found to be 100 nM. ∗ indicates significance compared to nontreated cells. (b) The dose-dependent effect of hesperidin (2.5, 5, 10, 20, and 40 *μ*g) alone and against rotenone-induced changes on cell proliferation. Values are presented as mean ± SD of four experiments in each group. Treatment with hesperidin alone (blue column) (2.5, 5, 10, 20, and 40 *μ*g) did not affect cell proliferation. Hesperidin (2.5, 5, 10, and 20 *μ*g) pretreatment dose dependently enhanced cell proliferation against rotenone toxicity.

Rotenone treatment (2.5, 5, 50, 100, and 200 nM for 24 h) of SK-N-SH cells induced a dose-dependent reduction in cell proliferation, with approximately 50% proliferation observed at 100 nM (Figure 1(a)). Hesperidin dose dependently (0.5, 5, 10, 20, and 40 *μ*g) attenuated the changes in cell proliferation induced by 100 nM rotenone (Figure 1(b)), with approximately 85% protection following treatment with 20 *μ*g hesperidin after 24 h.

### 3.2. Effect of Hesperidin on Rotenone-Induced ROS Formation

**Figure 2:**
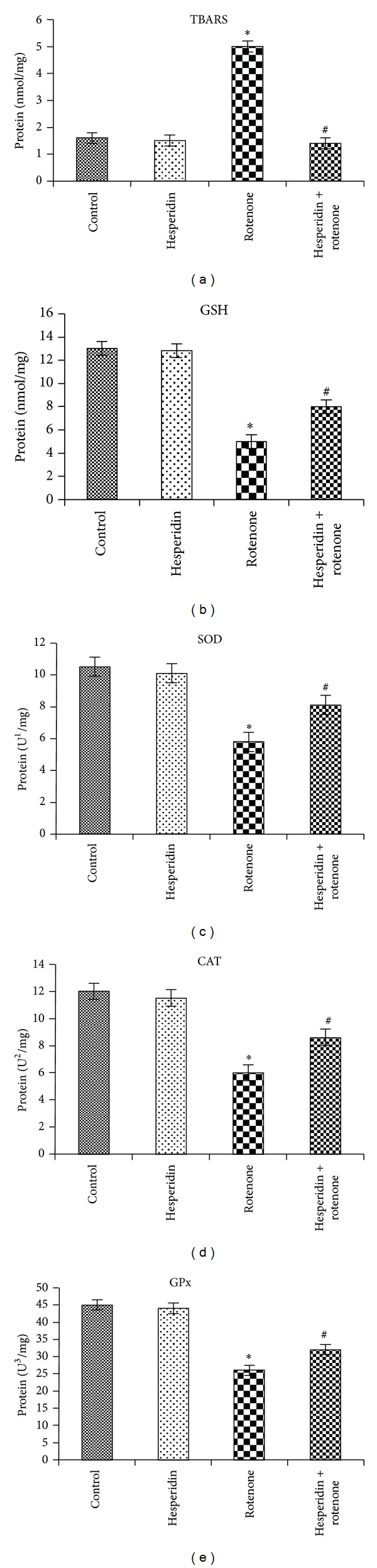
Effect of hesperidin (20 *μ*g) on rotenone (100 nM)-induced oxidative and antioxidative indices. Rotenone treatment significantly increased and decreased the levels of TBARS and GSH, respectively, as compared to control cells, while hesperidin pretreatment significantly decreased and enhanced the levels of TBARS and GSH as compared to rotenone alone treated cells (Figures 2(a) and 2(b)). Values are presented as mean ± SD of four experiments in each group. *P < 0.05 compared to control, and ^#^ *P* < 0.05 compared to rotenone group (DMRT). Rotenone treatment enhanced the activities of SOD, CAT, and GPx as compared to untreated cells, while hesperidin pretreatment significantly downregulated the activities of enzymatic antioxidants as compared to rotenone alone treated cells ((c), (d), and (e)). Values are given as mean ± SD of four experiments in each group. ^1^Enzyme concentration required for 50% inhibition of nitroblue tetrazolium reduction in 1 min. ^2^Micromoles of hydrogen peroxide consumed per minute. ^3^Micrograms of glutathione consumed per minute.

Figures 2(a) and 2(b) indicate the levels of TBARS and GSH in rotenone-treated SK-N-SH cells incubated with and without hesperidin. Rotenone treatment (100 nM) significantly increased the levels of TBARS parallel to decreased levels of GSH in SK-N-SH cells compared with nontreated cells. Pretreatment with hesperidin (20 *μ*g) to rotenone-treated cells significantly decreased the levels of TBARS and increased GSH levels significantly, compared to cells treated with rotenone alone. Figures 2(c), 2(d), and 2(e) elucidate the activities of SOD, catalase and GPx in rotenone-treated SK-N-SH cells incubated with and without hesperidin. Compared with untreated cells, rotenone (100 nM) treatment increased SOD, catalase and GPx activities in SK-N-SH cells (Figures 2(c), 2(d), and 2(e)). Pretreatment with hesperidin significantly decreased the activities of SOD, catalase, and GPx, compared to cells treated with rotenone alone.

### 3.3. Effect of Hesperidin on Intracellular ROS Generation

**Figure 3:**
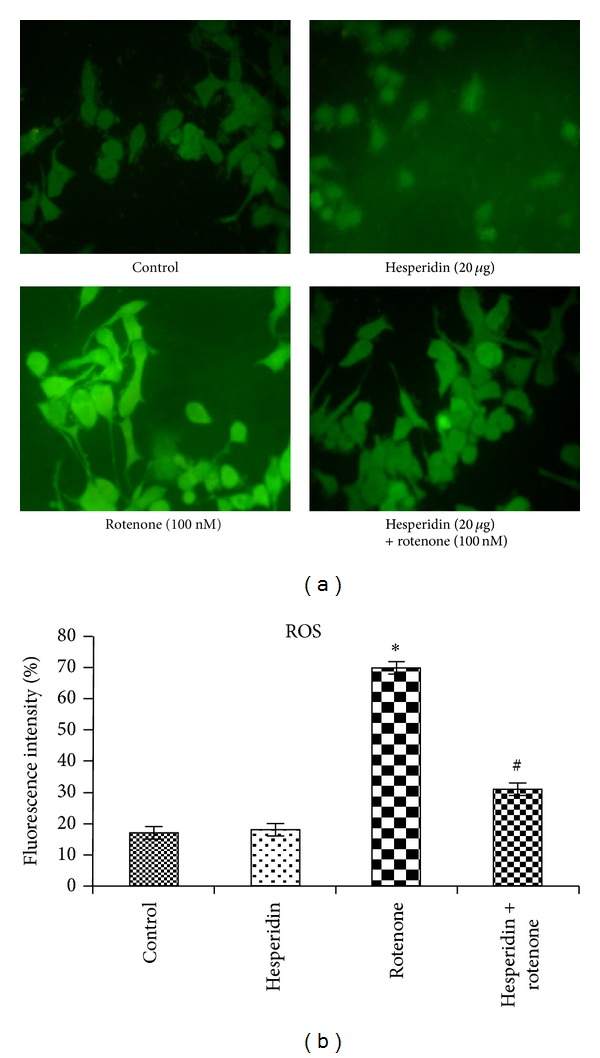
Effect of hesperidin on rotenone-induced ROS generation in SK-N-SH cells. (a) Microscopic images showing the preventive effect of hesperidin against rotenone-induced ROS generation by DCFDA staining. (b) Rotenone (100 nM) treatment significantly increased the levels of ROS as compared to control cells, while hesperidin (20 *μ*g) pretreatment significantly decreased the levels of ROS as compared to rotenone alone treated cells. Values are given as mean ± S.D. of four experiments in each group. *P < 0.05 compared to control, and ^#^ *P* < 0.05 compared to rotenone group (DMRT).

The formation of intracellular ROS was measured in terms of fluorescence by DCF (Figures 3(a) and 3(b)). Addition of rotenone (100 nM) to cells caused a significant increase (~411%) in DCF fluorescence. Pretreatment of the cells with hesperidin (20 *μ*g) lowered rotenone-induced free radical release as compared to rotenone-treated cells alone. No significant changes in ROS formation were detected in SK-N-SH cells treated with hesperidin alone.

### 3.4. Effect of Hesperidin on Rotenone-Induced ATP Depletion

**Figure 4:**
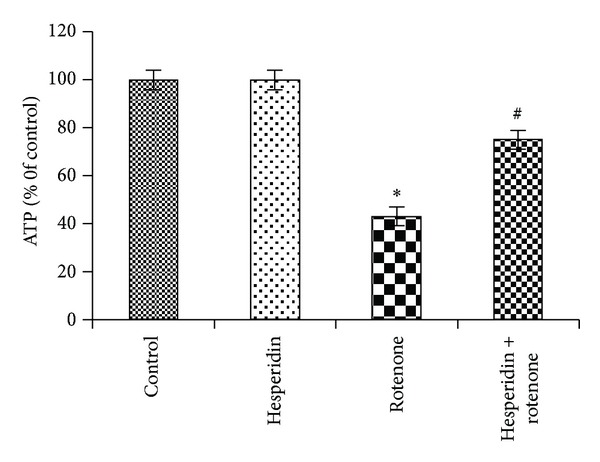
Measurement of intracellular ATP levels. Rotenone (100 nM) treatment significantly reduced the levels of ATP as compared to control cells, while hesperidin (20 *μ*g) pretreatment significantly enhanced the levels of ATP as compared to rotenone alone treated cells. Values are given as mean ± S.D. of four experiments in each group. *P < 0.05 compared to control, and ^#^ *P* < 0.05 compared to rotenone group (DMRT).

Rotenone treatment (24 hr) depleted cellular ATP levels (57 ± 4.8% at 100 nM) (Figure 4). Pretreatment with hesperidin increased ATP levels significantly compared to rotenone only treated cells. No significant changes in ATP levels were detected in SK-N-SH cells treated with hesperidin alone.

### 3.5. Effect of Hesperidin on Mitochondrial Membrane Potential (Δ*ψm*)

**Figure 5:**
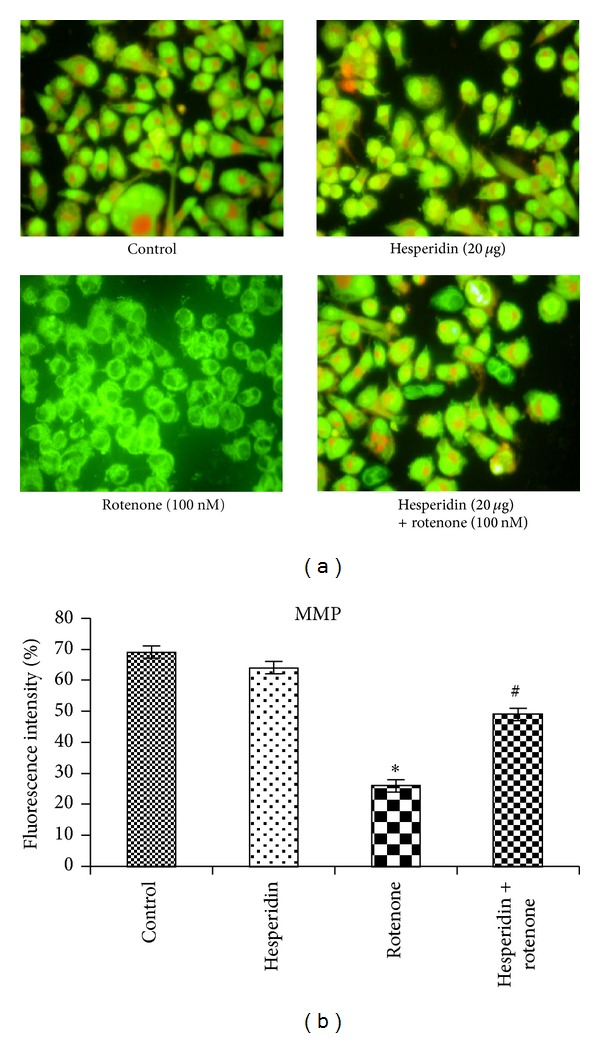
Alteration in mitochondrial membrane potential of control, hesperidin, and rotenone-treated SK-N-SH cells. Rotenone (100 nM) significantly decreased mitochondrial membrane potential, while hesperidin (20 *μ*g) pretreatment significantly increased MMP in rotenone-treated SK-N-SH cells ((a) and (b)). Values are given as mean ± S.D of four experiments in each group. *P < 0.05 compared to control, and ^#^ *P* < 0.05 compared to rotenone groups (DMRT).

Figures 5(a) and 5(b) show the mitochondrial membrane potential (Δ*ψm*) measured by determining the red/green fluorescence ratio in the presence of Rh-123. Cells treated with 100 nM rotenone resulted in significant dissipation of  Δ*ψm*. The average green fluorescence ratio was increased as a result of rotenone treatment as compared to untreated controls. Pretreatment with hesperidin to rotenone-treated cells displayed much higher red/green fluorescence, indicating a polarized state of mitochondrial membrane as compared to rotenone treatment alone.

### 3.6. Effect of Hesperidin on Apoptotic Markers

**Figure 6:**
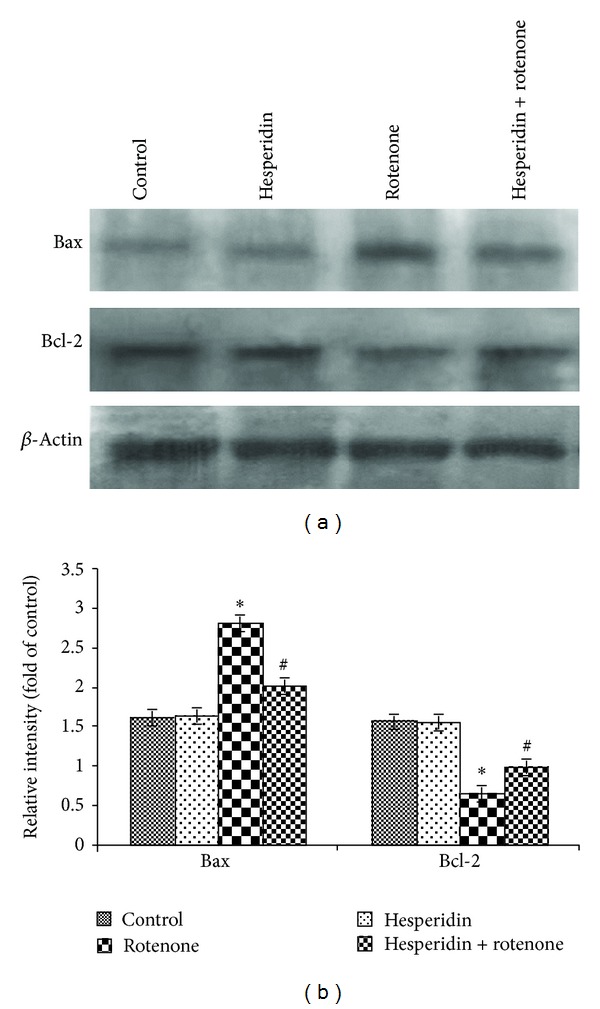
Effect of hesperidin on rotenone-induced Bax and Bcl-2 expressions in SK-N-SH cells. Rotenone (100 nM) significantly enhanced the expression of Bax and diminished the expression of Bcl-2, while hesperidin (20 *μ*g) pretreatment significantly diminished the expression of Bax and elevated the expression of Bcl-2 in rotenone-treated SK-N-SH cells ((a) and (b)). Western blot data are quantified by using *β*-actin as an internal control, and the values are expressed as arbitrary units and given as mean ± SD of four experiments in each group. *P < 0.05 compared to control, and ^#^ *P* < 0.05 compared to rotenone alone treated group.

**Figure 7:**
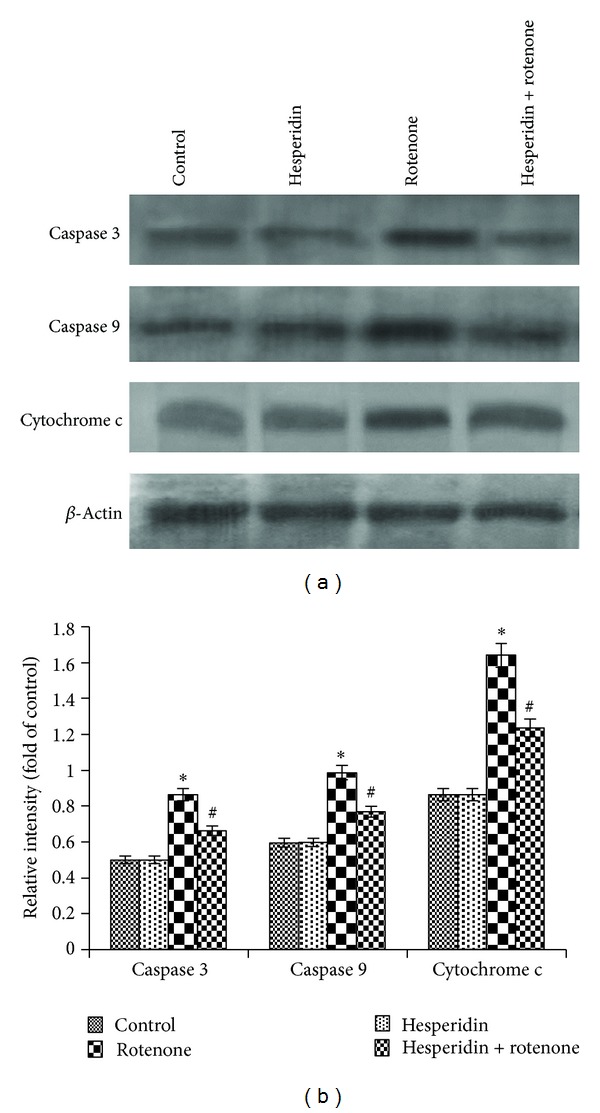
Effect of hesperidin on rotenone-induced cyt c and caspases 3 and 9 expressions in SK-N-SH cells. Rotenone (100 nM) significantly enhanced the expressions of cyt c, caspases 3 and 9, while hesperidin (20 *μ*g) pretreatment significantly diminished the expressions of cyt c, caspases 3 and 9 in rotenone-treated SK-N-SH cells ((a), (b), and (c)). Western blot data are quantified by using *β*-actin as an internal control, and the values are expressed as arbitrary units and given as mean ± SD of four experiments in each group. *P < 0.05 compared to control, ^#^ *P* < 0.05 compared to rotenone groups.

Rotenone treatment (100 nM) decreased the protein expression levels of antiapoptotic B-cell CLL/lymphoma 2 (Bcl-2) and increased the protein expression levels of proapoptotic Bcl-2-associated X protein (Bax) (Figures 5(a) and 5(b)). Hesperidin pretreatment (20 *μ*g) attenuated the rotenone-induced reduction in Bcl-2 expression and increased expression of Bax (Figures 6(a) and 6(b)). We also examined the effect of hesperidin on the protein expression of cyt c and caspases 3 and 9. Treatment with rotenone (100 nM) for 24 h increased the expression of cyt c, caspases 3 and 9 at the protein level (Figures 7(a) and 7(b)). Hesperidin pretreatment significantly ameliorated the rotenone-mediated increase in cyt c, caspases 3 and 9 protein expression levels (Figures 7(a) and 7(b)).

## 4. Discussion

Our results show that rotenone is cytotoxic to SK-N-SH neuroblastoma cells in line with previous studies. However, our group is the first to show that hesperidin can attenuate the toxic cascade in rotenone-treated cells. Reduction of the tetrazolium salt MTT to a blue formazan product is widely used for assessing cellular proliferation. The reduction is mainly catalyzed by dehydrogenases localized in the mitochondria of proliferating cells [23]. Optimal mitochondrial function is a determinant of the proliferation and hence overall viability of these cells by playing a central role in regulating apoptotic cell death signalling by controlling cellular energy metabolism, contribution of reactive oxygen species (ROS) formation, and release of apoptotic factors into the cytosol [24]. Data obtained from the MTT assay in the present study suggest a direct neuroprotective action of hesperidin against rotenone-mediated mitochondrial dysfunction.

Our data also demonstrate that rotenone treatment can stimulate the generation of intracellular ROS in SK-N-SH cells, as corroborated with previous studies [25, 26]. Inhibition of respiratory complex I by rotenone causes electrons to accumulate within respiratory chain components. These electrons can be added directly to oxygen molecules to produce O_2_^−^ [27, 28]. The mitochondria are the principle intracellular sources of ROS and also the major targets of oxidative stress [29]. Hesperidin treatment significantly reduced ROS generation in rotenone-treated cells, which might be due to ROS scavenging property of hesperidin [30] and transition metal ions chelating properties [31]. Additionally, other potential mechanisms such as the ability of hesperidin to enhance glutathione content could be involved in its protective effect on rotenone-induced oxidative stress generation.

When cells were incubated with rotenone, we found increased levels of TBARS, indicating overproduction of free radicals along with glutathione depletion, which is corroborated with previous studies [7]. Our results show that the increase in the enzymatic activities of SOD, GPx, and CAT in neuroblastoma cells incubated with rotenone alone is likely due to a response towards increased ROS generation following rotenone treatment. Cassarino et al. [32] reported that complex I inhibition induces SOD activity in the brain tissue. Increment in GPx activity in rotenone alone treated cells indicated that there was an increase in the concentration of lipid peroxides and/or H_2_O_2_. Reduced GSH levels may also account for the increase in GPx activity [7].

In the present study, ATP levels were significantly reduced in rotenone-treated cells alone as compared to nontreated controls, which corroborates with previous studies [26, 33]. Since ATP is necessary for downstream events in the apoptotic cascade [34], the depletion of the intracellular ATP supply may not be expected to induce apoptosis. One study showed that both rotenone and 2-deoxyglucose caused similar ATP depletion. However, only rotenone exposure was toxic, suggesting that rotenone toxicity resulted from additional mechanisms such as oxidative damage [26, 35]. Similarly, mitochondrial ETC inhibitors such as rotenone deplete ATP and induce apoptosis, while oxidative phosphorylation inhibitors, which inhibit ATP synthesis without inhibiting the ETC, fail to induce apoptosis despite depleting ATP to the same extent as ETC inhibitors in dopaminergic neuronal cells. Therefore, intracellular ATP depletion *per se* is not sufficient for apoptosis induction. Rather, ROS production plays an essential role in apoptosis induced by mitochondrial ETC inhibition. Pretreatment with hesperidin partially attenuated rotenone-induced ATP deficiency.

It was reported by several groups that complex I inhibition by rotenone may result in the opening of mitochondrial permeability transition pores (PTP), which induces a specific conformational change of complex I and massive production of ROS [36–38]. Increased levels of ROS within the mitochondria are known to cause further mitochondrial membrane depolarization and further release of ROS. Due to rotenone treatment, mitochondrial membrane potential is reduced, leading to increased mitochondrial permeability and results in the enhanced release of cyt c from the mitochondria, which triggers activation of caspases 3 and 9 culminating with cell death [39]. cyt c is not only an important electron carrier in the mitochondrial respiration chain, but also a death messenger in the cytosol, which forms apoptosome complexes with Apaf-1, dATP, and caspases 9 and 3 [40].

The efflux of the cyt c from the mitochondria is also regulated by BAD protein [41]. In an apoptotic cell, BAD protein displaces Bax and binds to the antiapoptotic members Bcl-2 and Bcl-xL [41]. Bax and Bcl-2 are involved in the regulation of caspase 3 mediated apoptosis [42]. Numerous studies have shown that Bcl-2, as a negative regulator of cell death in the Bcl-2 family members, protects cells against apoptosis induced by various stimuli in a wide variety of cell types [43], whereas Bax is a positive regulator of cell death which promotes or accelerates cell death. It was reported that rotenone induces Bax expression in dopaminergic neuronal cell line MND9 [44]. Elevations in proapoptotic proteins, such as Bax, are believed to stimulate mitochondrial generation of ROS and contribute to neuronal cell death in neurodegenerative diseases. Moreover, overexpression of Bcl-2 disrupts the proapoptotic proteins of Bax and prevents the mitochondrial release of cyt c, thereby inhibiting the activation of caspases and ultimately apoptosis [45, 46]. The ratio of Bcl-2 to Bax determines the survival or death of neurons following an apoptotic stimulus. Administration of hesperidin prevents the loss of  Δ*ψm*, increases the mitochondrial permeability, and prevents the release of cyt c from the mitochondria, thereby inhibiting caspases 3 and 9, thus restoring the imbalance in the expression profiles of Bax and Bcl-2, and preventing cell death. Moreover, overexpression of Bcl-2 disrupts the proapoptotic proteins of Bax and prevents the mitochondrial release of cyt c, thereby inhibiting the activation of caspases, and apoptosis [45, 46].

Although the exact neuroprotective mechanism of action of hesperidin remains unclear, it is likely to demonstrate both antioxidant and cell signalling properties. Our data is in line with previous studies showing that hesperidin therapy could significantly attenuate ROS formation by reducing the levels of TBARS and restored antioxidant enzyme activity and GSH to physiological levels in the brain [47] and cultured PC12 cells [48]. Another study showed that hesperidin treatment could reduce stress-induced anxiety, impaired locomotor activity, and mitochondrial dysfunction in mice by modulating the nitrergic pathway [49]. Hesperidin also protected cortical neurons from ROS-mediated injury by activation of the prosurvival Akt and ERK1/2 signalling pathways [50]. These pathways are involved in the inhibition of the release of proapoptotic proteins such as apoptosis signal-regulating kinase 1 (ASK1), BAD, and caspases 3 and 9, suggesting that the neuroprotective effects of hesperidin may be due to its effect on a yet unidentified receptor [50]. Moreover, hesperidin can also protect against amyloid-beta- (A*β*-) associated neurotoxicity, and glutamate-induced excitotoxicity [13]. A more recent study showed that hesperidin treatment could reduce cerebral damage due to induced stroke in the rat brain due to the reduction of free radicals and associated neuroinflammation [51]. The results of our present study suggest that hesperidin attenuates neuronal damage induced by rotenone by reducing oxidative stress, mitochondrial dysfunction, and ameliorating apoptosis. These findings may have important implications in the use of hesperidin for the prevention of PD. However, further research involving various animal models and clinical trials is needed to validate hesperidin as a new therapeutic agent.
