# EXPRESSION OF CONCERN: Neuroprotective Effects of Hesperidin, a Plant Flavanone, on Rotenone‐Induced Oxidative Stress and Apoptosis in a Cellular Model for Parkinson’s Disease

**DOI:** 10.1155/omcl/9828315

**Published:** 2026-07-13

EXPRESSION OF CONCERN: K. Tamilselvam, N. Braidy, T. Manivasagam, et al., “Neuroprotective Effects of Hesperidin, a Plant Flavanone, on Rotenone‐Induced Oxidative Stress and Apoptosis in a Cellular Model for Parkinson’s Disease,” *Oxidative Medicine and Cellular Longevity*, vol. 2013 (2013). https://doi.org/10.1155/2013/102741.

This Expression of Concern is for the above article, published online on 15 August 2013 in Wiley Online Library (onlinelibrary.wiley.com), and has been issued following the agreement of the Journal’s Editor‐in‐Chief, Jeannette Vasquez‐Vivar; and John Wiley & Sons Ltd.

The Expression of Concern has been issued due to the investigation of the concerns flagged by a third party on PubPeer [1]. The concerns relate to the similarity of the panels showing SK‐N‐SH cells from the control and hesperidin groups in Figure 5a, as well as the reuse of the image of cells treated with 100 nM rotenone in Figure 3a in another publication by the author group, where it was used to represent a different experimental outcome [2]. The authors were contacted on several occasions and asked to provide the raw data supporting the affected figures; however, the publisher did not receive the raw data requested. In light of this, the journal could not verify the validity of these images and could not definitively conclude that the above‐mentioned concerns affect the overall conclusions of the article. Therefore, the journal has decided to issue an Expression of Concern to inform and alert readers. The authors have been notified.
